# Discriminating between Glaucoma and Normal Eyes Using Optical Coherence Tomography and the ‘Random Forests’ Classifier

**DOI:** 10.1371/journal.pone.0106117

**Published:** 2014-08-28

**Authors:** Tatsuya Yoshida, Aiko Iwase, Hiroyo Hirasawa, Hiroshi Murata, Chihiro Mayama, Makoto Araie, Ryo Asaoka

**Affiliations:** 1 Department of Ophthalmology, The University of Tokyo, Tokyo, Japan; 2 Tajimi Iwase Eye Clinic, Tajimi, Japan; 3 Kanto Central Hospital of the Mutual Aid Association of Public School Teachers, Tokyo, Japan; Saitama Medical University, Japan

## Abstract

**Purpose:**

To diagnose glaucoma based on spectral domain optical coherence tomography (SD-OCT) measurements using the ‘Random Forests’ method.

**Methods:**

SD-OCT was conducted in 126 eyes of 126 open angle glaucoma (OAG) patients and 84 eyes of 84 normal subjects. The Random Forests method was then applied to discriminate between glaucoma and normal eyes using 151 OCT parameters including thickness measurements of circumpapillary retinal nerve fiber layer (cpRNFL), the macular RNFL (mRNFL) and the ganglion cell layer-inner plexiform layer combined (GCIPL). The area under the receiver operating characteristic curve (AROC) was calculated using the Random Forests method adopting leave-one-out cross validation. For comparison, AROCs were calculated based on each one of the 151 OCT parameters.

**Results:**

The AROC obtained with the Random Forests method was 98.5% [95% Confidence interval (CI): 97.1–99.9%], which was significantly larger than the AROCs derived from any single OCT parameter (maxima were: 92.8 [CI: 89.4–96.2] %, 94.3 [CI: 91.1–97.6] % and 91.8 [CI: 88.2–95.4] % for cpRNFL-, mRNFL- and GCIPL-related parameters, respectively; P<0.05, DeLong’s method with Holm’s correction for multiple comparisons). The partial AROC above specificity of 80%, for the Random Forests method was equal to 18.5 [CI: 16.8–19.6] %, which was also significantly larger than the AROCs of any single OCT parameter (P<0.05, Bootstrap method with Holm’s correction for multiple comparisons).

**Conclusions:**

The Random Forests method, analyzing multiple SD-OCT parameters concurrently, significantly improves the diagnosis of glaucoma compared with using any single SD-OCT measurement.

## Introduction

Early diagnosis of glaucoma is essential since glaucomatous visual field (VF) damage is irreversible. In glaucoma, it is clinically important to evaluate structural changes at the optic nerve head [1]–[3] and damage to the retinal nerve fiber layer (RNFL) around the optic disc [4]–[6], because *measureable* structural transformation can precede *measurable* VF damage.

The development of spectral domain optical coherence tomography (SD-OCT) has enabled imaging scans of the macular retinal nerve fiber layer (mRNFL), the macular ganglion cell layer-inner plexiform layer combined (GCIPL) [7], [8] and the circumpapillary RNFL (cpRNFL), which are all reported to be damaged early on in the glaucomatous disease process. [7], [9]–[15] Many previous studies have investigated the performance of SD-OCT to diagnose glaucoma using thickness measurements of these structures, [7], [9]–[15] usually by evaluating each measurement individually. However, damage to these layers occurs concurrently and in characteristic patterns. [16], [17] Consequently, to improve the diagnostic ability of SD-OCT for glaucoma, several mathematical models have been constructed to analyze these different structural measurements in combination. [18]–[20] For example, Mwanza et al. analyzed multiple SD-OCT parameters using a logistic regression model [18], while Burgansky-Eliash et al. adopted a support vector machine classifier to analyze combined measurements from time domain (TD)-OCT [19]; both models were able to successfully improve the diagnosis of glaucoma. Moreover, a recent study demonstrated the usefulness of decision trees, analyzing multiple OCT parameters, to discriminate between glaucoma and normal eyes [20].

The decision tree classifier, a ‘pattern recognition’ method, is a tree-like representation of a finite set of if-then-else rules [21]. One well-known drawback of the method is the problem of ‘overfitting’, which influences diagnostic accuracy. [22] Indeed, in a previous study by our group, the decision tree method was found to perform less accurately than the Random Forests method at discriminating between perimetric and preperimetric glaucoma eyes. [23] The Random Forests method [24], [25], originally proposed by Breiman in 2001, is more robust to the overfitting problem, because it is composed of many different decision trees. Each one developed using a different sample of data and as a result, the prediction accuracy tends to be far improved over the decision tree method [26].

Previous reports have indicated that the Random Forests method is more accurate than other machine learning methods, [27], [28] and can cope with inter-correlation between multiple explanatory variables, since each predictor is selected randomly for each stage of the learning process, and thus each predictor has less opportunity to compete against correlated predictors, [29] (unlike standard regression approaches). This particular quality makes the Random Forests method especially useful for diagnosing glaucoma based on multiple OCT parameters, namely thickness measurements of the cpRNFL, the mRNFL and the GCIPL, which are correlated. Indeed the Random Forests method has been used to explore interactions between different explanatory variables. [29]–[31] Furthermore, there is considerable overlap in the distributions of these OCT measurements between normal and glaucoma eyes [32], [33], and hence, a simple comparison of a measured OCT parameter with a normative database will have rather limited diagnostic ability.

The purpose of the current study was to investigate the ability of the Random Forests method to discriminate between normal and glaucoma eyes by concurrently analyzing cpRNFL, mRNFL and GCIPL measurements, and compare the results with those obtained using individual measurements of these structures.

## Materials and Methods

The study was approved by the Research Ethics Committee of the Graduate School of Medicine and Faculty of Medicine at the University of Tokyo. Written consent was given by the patients for their information to be stored in the hospital database and used for research. This study was performed according to the tenets of the Declaration of Helsinki.

### Subjects

All of the following measurements were conducted either at the University of Tokyo Hospital or the Tajimi Iwase eye clinic. Subjects underwent complete ophthalmic examinations, including biomicroscopy, gonioscopy, intraocular pressure measurement, funduscopy, refraction and corneal radius of curvature measurements using an automatic refractometer (ARK-900; NIDEK, Tokyo, Japan), best-corrected visual acuity measurements and axial length (AL) measurements (IOL Master; Carl Zeiss Meditec, Dublin, CA), as well as imaging with SD-OCT and VF testing.

The glaucoma group in this study comprised 126 eyes of 126 subjects with open angle glaucoma (OAG) who were enrolled between January 2009 and March 2010. Glaucoma was diagnosed when the following findings were present: 1) presence of apparent glaucomatous changes in the optic nerve head (ONH), according to a stereo-fundus photograph, such as a rim notch with a rim width ≤0.1, a vertical cup-to-disc ratio of >0.7 and/or a RNFL defect (with its edge at the ONH margin greater than a major retinal vessel) diverging in an arcuate or wedge shape, as confirmed by a panel of glaucoma specialists (A.I., M.A., and R.A.); 2) presence of glaucomatous VF defects, compatible with glaucomatous ONH changes, fulfilling at least one of Anderson-Patella’s criteria, i.e., a cluster of ≥3 points (3 non-edge points if VF was tested with HFA 30-2 test program) in the pattern deviation plot in a single hemifield (superior/inferior) with P<0.05, one of which must have been P<0.01, a glaucoma hemifield test result outside of normal limits, or an abnormal pattern standard deviation with P<0.05 [34]; and 3) absence of other systemic or ocular disorders including a shallow peripheral anterior chamber that could affect the ONH and VF including intraocular surgeries or refractive surgeries (except for uneventful intraocular lens implantation). Patients aged 20 years or older and eyes with refractive error ≥−6.0 D and <3.0 D were included. If both eyes of a subject fulfilled the inclusion criteria, the eye with a better data quality factor in the SD-OCT examination was included in the study.

The normal group consisted of 84 eyes of 84 normal subjects. Inclusion criteria were no abnormal findings except for clinically insignificant senile cataract on biomicroscopy, gonioscopy and funduscopy, and no history of ocular diseases that could affect the results of SD-OCT examinations, such as diabetic retinopathy or age-related macular degeneration. Other inclusion criteria were age ≥20 years old, spherical equivalent refractive error ≥−6.0 D and <3.0 D, and normal VF test results according to Anderson-Patella’s criteria. [34] Eyes with anomalous discs including tilted discs [35] were cautiously excluded.

VF testing was performed, within 3 months of the SD-OCT examination, using the Humphrey Field Analyzer (HFA, Carl Zeiss Meditec) with the SITA Standard strategy and the Goldmann size III target. VFs of the normal group were measured using the 24-2 test program while VFs of the glaucoma group were measured using either the 24-2 or 30-2 test program. Near refractive correction was used as necessary. Unreliable VFs defined as fixation losses greater than 25%, or false-positive responses greater than 15% were excluded. [36] All of the participants had previous experience in undergoing VF examinations.

### SD-OCT data acquisition

SD-OCT data were obtained using the 3D OCT-1000 (Topcon Corp., Tokyo, Japan) in the normal group while patients in the glaucoma group underwent imaging using either the 3D OCT-1000 (68 eyes) or 3D OCT-2000 (Topcon Corp., Tokyo, Japan) (58 eyes). The results obtained with 3D OCT-1000 are interchangeable with those from 3D-OCT 2000, but scanning speed in the latter instrument is 90% faster. All SD-OCT measurements were performed after pupil dilation with 1% tropicamide.

SD-OCT imaging was performed using the raster-scan protocol in which data were obtained in a 6.0×6.0 mm square area (512×128 pixels), centered on the point of fixation, in 2.5 seconds with 3D-OCT 1000, or in a 7.0×7.0 mm square area, centered on the point of fixation, in 1.3 seconds with 3D-OCT 2000. The magnification effect was corrected according to the manufacturer-provided formula (modified Littman’s equation) [37], [38], which is based on measured refractive error, corneal radius, and axial length. Registration of fundus photographs and OCT images was automatically confirmed using an OCT projection image (generated from 3D-OCT data by summing different retinal depth levels) and localization of major retinal vessels.

A similar raster scan was then performed centered on the disc. In this study, 7 points were manually determined on the optic disc edge in a color fundus photograph simultaneously obtained by the non-mydriatic fundus camera function. The optic disc center was determined in fundus photographs as the barycenter of the closed spline curve fitted to the manually determined 7 points; the point was then extrapolated in all OCT images thereafter.

Data obtained during apparent eye movements were discarded and re-examined, and those with the best quality factor (given by the SD-OCT apparatus based on signal intensity) were used in each area. Further, images influenced by involuntary blinking or saccade, indicated by breaks or shifting of the vessel or disc images or a straight line across the fundus OCT image, respectively, or those with quality factor <60% were excluded.

### Analysis of SD-OCT data

**Figure 1:**
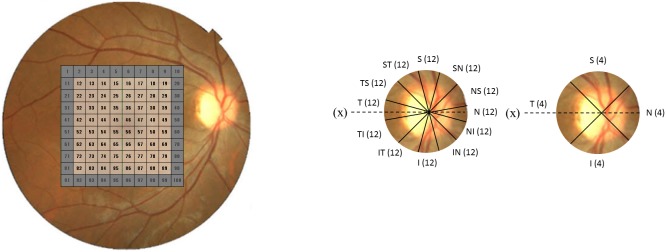
Macular area centered on the fovea (6×6 mm) and mRNFL and GCIPL grids, and the RNFL thickness along a 3.4-mm diameter circle centered on the disc barycenter. **a**: Macular area centered on the fovea (6×6 mm) and mRNFL and GCIPL grids. The mRNFL and GCIPL grids at the most outer circumference (sectors 1–10, 11, 20, 21, 30, 31, 40, 41, 50, 51, 60, 61, 70, 71, 80, 81, 90–100: colored in grey) were excluded from analysis. If a subject’s left eye was measured, recorded data were mirror-imaged to reflect those in the right eye. **b**: RNFL thickness along a 3.4-mm diameter circle centered on the disc barycenter. T: temporal, TS: temporal-superior, ST: superior-temporal, S: superior, SN: superior-nasal, NS: nasal-superior, N: nasal, NI: nasal-inferior, IN: inferior-nasal, I: inferior, IT: inferior-temporal, TI: temporal-inferior.

In the macular area, the fovea was automatically identified in the acquired OCT image as the pixel with the thinnest retinal thickness close to the fixation point, and the square area centered on the fovea was first set and divided into 10×10 equally-sized grids. The analysis area consists of the inner 8×8 grids, excluding the outermost units (grids 1–10, 11, 20, 21, 30, 31, 40, 41, 50, 51, 60, 61, 70, 71, 80, 81, 90–100: see **Figure 1a**). In some of the OCT images obtained with 3D-OCT 1000, the analysis area was found to have run off the edge of the 6.0×6.0 mm data acquisition area, and such OCT images were excluded. The inner boundary of the retina was the surface of the RNFL while the outer boundary was retinal pigmentary epithelium. RNFL and GCIPL were automatically segmented [39] and confirmed on all B-scan images by an experienced examiner (H.H), and the thicknesses of the mRNFL and GCIPL were determined at each pixel. The thickness of each retinal layer in each grid was calculated as the mean value of the thickness over all pixels contained in the grid and the mean thickness of each layer over the whole analysis area (4.8×4.8 mm area centered on the fovea) (mean mRNFL or GCIPL), superior hemiretina (2.4×4.8 mm area) and inferior hemiretina were also calculated.

RNFL thickness along a 3.4 mm diameter circle centered on the disc barycenter was also obtained from the raster scan data and cpRNFL thickness was calculated for the whole circumference of the circle (360°- cpRNFL), the four cpRNFL sectors each accounting for 90° (temporal, superior, nasal and inferior quadrant; 90°-cpRNFL) and 12 cpRNFL sectors each accounting for 30° (30°-cpRNFL, respectively); see **Figure 1b**.

The Random Forests method was used to discriminate glaucoma eyes from normal eyes using the 151 OCT measurements listed in **Table 1**. In this study, 10,000 decision trees were grown in the Random Forests. The leave-one-out cross validation method was performed and the area under the receiver operating characteristic curve (AROC) was calculated by changing the cut-off value of the probability of glaucoma (as indicated by the proportion of 10,000 decision tree votes in the Random Forests). [40] In leave-one-out cross validation, a single eye was used as validation data and the remaining eyes were used as training data; this procedure was repeated such that each eye in the original sample was used only once as validation data. In other words, for each individual, only the data from all other subjects (n = 209 in 210) was used to produce a diagnosis. For comparison, AROCs were also derived using the 151 individual OCT measurements.

**Table 1:** SD-OCT parameters used in the Random Forests method.

SD-OCT parameters	
cpRNFL(17 in total)	Total4 quadrants (superior, temporal, nasal, inferior quadrant)12 (temporal, temporal-superior, superior-temporal, superior, superior-nasal, nasal-superior, nasal, nasal-inferior, inferior-nasal, inferior, inferior-temporal, temporal-inferior) sectors
mRNFL(67 in total)	Total2 sectors (superior and inferior hemiretina)64 grid
GCIPL(67 in total)	Total2 sectors (superior and inferior hemiretina)64 grid

SD-OCT: spectral domain optical coherence tomography, cpRNFL: circumpapillary retinal nerve fiber layer (RNFL), mRNFL: macular RNFL, GCIPL: ganglion cell layer- inner plexiform layer combined, P value: comparison between glaucoma and normal groups (unpaired t-test for numerical data and chi-square test for categorical data).

Using all subjects (n = 210), significant (P<0.05) OCT parameters in the Random Forests were calculated by randomly permuting the variable at each decision tree and observing the decrease in the number of correct classifications [25].

All statistical analyses were carried out using the statistical programming language R (ver. 2.15.1, The R Foundation for Statistical Computing, Vienna, Austria) and Medcalc version 11.4.2.0; MedCalc statistical software, Mariakerke, Belgium). The R package “randomForest” was used to carry out the Random Forests analysis. The AROC was used to evaluate the clinical usefulness of each classifier, as suggested in a previous paper. [41] Comparison of multiple AROCs was carried out using DeLong’s method. [42] Holm’s method [43], [44] was used to correct P values for the problem of multiple testing. Partial AROCs with specificity above 80 and 90% were also compared using the bootstrap method. [45], [46] The optimum cut-off point in the ROC was calculated using Youden’s method [47].

## Results

Subject characteristics are given in **Table 2**. Thickness of the 360°-cpRNFL, mean mRNFL and GCIPL were significantly smaller in the glaucoma group compared with the normal group (P<0.001, unpaired t-test). Significant inter-group differences in MD and refractive error were also observed (P<0.001, P = 0.002, respectively, unpaired t-test). There were no significant differences between right/left eye and male/female ratios (P = 0.067 and 0.89, respectively; chi-square test).

**Table 2:** Characteristics of the study participants.

	Glaucoma group	Normal group	p value
Eye (right/left)	68/58	65/19	0.067
Age, y (mean ± sd)	60.1±13.1	52.6±15.6	<0.001
Gender (male/female)	53/73	47/37	0.89
MD, dB (mean ± sd)[range]	–5.6±5.2[–23.2 to 1.8]	–0.4±1.3[–3.5 to 2.1]	<0.001
Refractive error, diopters(mean ± sd) [range]	–0.79±1.3[–2.9 to 2.0]	–0.23±1.2[–2.8 to 2.6]	0.002
cpRNFL, µm(mean ± sd) [range]	80.3±13.6[49.0 to 114.3]	102.4±8.3[81.9 to 121.5]	<0.001
mRNFL, µm(mean ± sd) [range]	24.8±7.0[4.9 to 43.4]	36.0±3.9[28.0 to 46.3]	<0.001
GCIPL, µm(mean ± sd) [range]	61.4±5.7[43.7 to 74.8]	70.1±4.3[61.2 to 82.1]	<0.001

sd: standard deviation, MD: mean deviation, cpRNFL: circumpapillary retinal nerve fiber layer (RNFL), mRNFL: macular RNFL, GCIPL: ganglion cell layer and inner plexiform layer combined.

The Random Forests classifier in this study was built using 10,000 decision trees – with this number of trees the error rate was saturated and thus increasing the number of trees in the forest will not improve diagnostic accuracy (results not shown).

**Figure 2:**
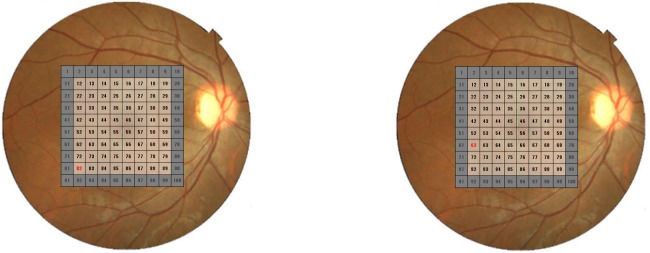
The mRNFL and GCIPL grid giving the largest AROC. **a**: Largest AROC (94.3 [CI: 91.1–97.6] %) was obtained using the mRNFL grid with reference 82 (highlighted red). **b**: Largest AROC (91.2 [CI: 87.3–95.1] %) was obtained using the GCIPL grid with reference 62 (highlighted red). OCT: optical coherent tomography, AROC: area under the receiver operating characteristic curve, mRNFL: macular retinal nerve fiber layer, GCIPL: ganglion cell layer -inner plexiform layer combined. CI: 95% confidence interval.

**Figure 3:**
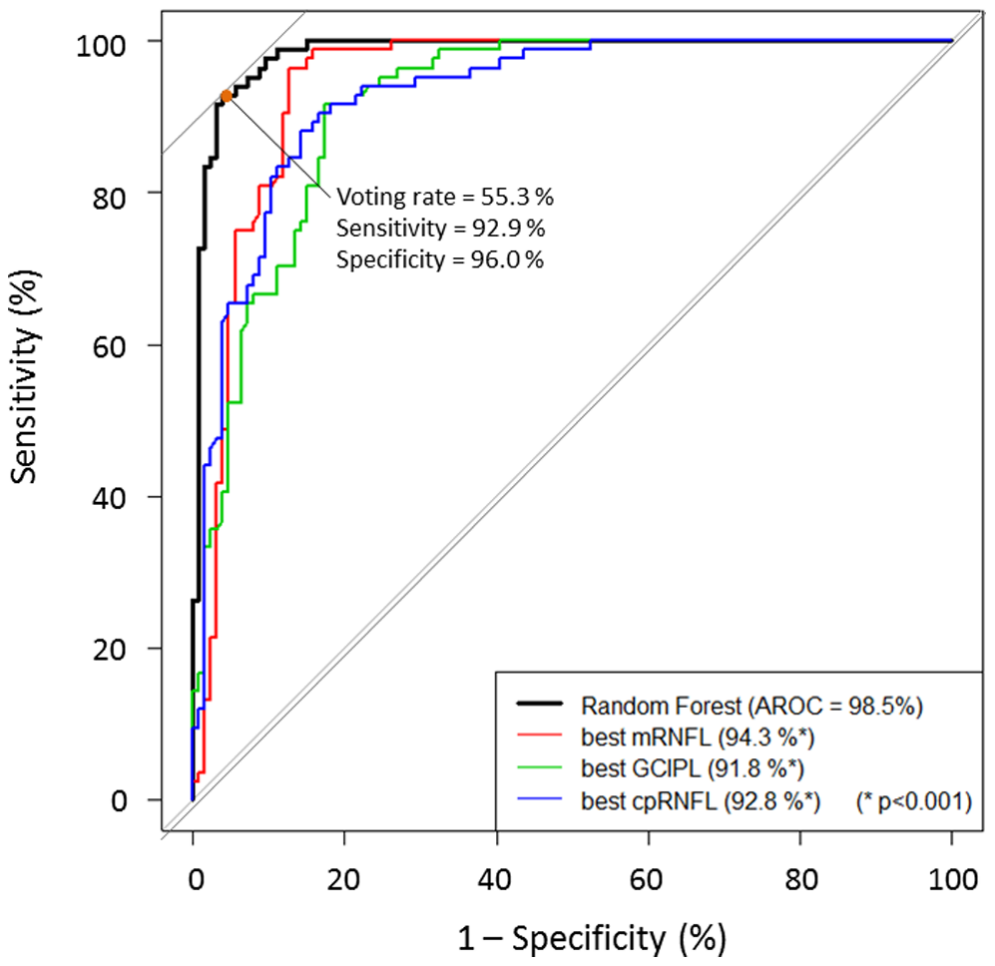
Receiver operating characteristic (ROC) curves obtained with the Random Forests method and the best performing cpRNFL-, mRNFL- or GCIPL-related parameters. The area under the ROC (AROC) with the Random Forests method was significantly larger than those with the individual cpRNFL-, mRNFL- or GCIPL-related parameters (P<0.001, DeLong’s method. All P values were significant after correction for multiple testing using Holm’s method [43], [44]). The orange dot on the ROC of the Random Forests method represents the optimal threshold; the voting rate at this point was 55.3%, giving a sensitivity of 92.9% with specificity equal to 96.0%. [47] cpRNFL: circumpapillary retinal nerve fiber layer (RNFL), mRNFL: macular RNFL, GCIPL: ganglion cell layer- inner plexiform layer combined.

The AROCs obtained using thickness measurements of 360°-cpRNFL, superior and 90°-cpRNFL, and 12 30°-cpRNFL, mean mRNFL and GCIPL, superior and inferior hemiretina mRNFL and GCIPL are shown in **Table 3**. The AROCs obtained using each of the 64 grids for mRNFL and GCIPL ranged between 52.8 [CI 44.9–60.7] to 94.3 [CI: 91.1–97.6] %, and between 56.3 [CI: 48.4–64.2] to 91.2 [87.3–95.1] %, respectively (**Table 3**); the largest AROC for an mRNFL grid (grid number 82) was 94.3 [CI: 91.1–97.6] % and the largest AROC for a GCIPL grid (grid number 62) was 91.2 [CI: 87.3–95.1] %; see **Figure 2a and b**. Among cpRNFL-, mRNFL- and GCIPL-related OCT parameters, the largest AROCs were 92.8 [CI: 89.4–96.2] % (360°-cpRNFL), 94.3 [CI: 91.1–97.6] % (grid number 82, mRNFL) and 91.8 [CI: 88.2–95.4] % (lower hemiretina GCIPL); see **Table 3**. The AROC obtained using the Random Forests method was 98.5 [CI: 97.1–99.9] %, which was significantly larger than any AROCs obtained using a single OCT parameter. (P<0.001, DeLong’s method after correction of P values for multiple testing using Holm’s method [43], [44]); see **Figure 3**. The optimum discrimination of the Random Forests method, using Youden’s method, was at a sensitivity of 92.9% and specificity of 96.0%; this corresponded to a cut-off voting rate of 55.3%, where a proportion larger than this indicates a diagnosis of glaucoma [47].

**Table 3:** AROCs with summary SD-OCT measures.

SD-OCT parameter	AROC [CI] (%)
total cpRNFL	92.8 [89.4–96.2]
temporal quadrant cpRNFL	73.6 [67.0–80.2]
superior quadrant cpRNFL	82.6 [77.2–88.0]
nasal quadrant cpRNFL	78.7 [72.5–84.8]
inferior quadrant cpRNFL	90.9 [87.0–94.8]
12 sector cpRNFL (temporal)	61.3 [53.8–68.9]
12 sector cpRNFL (temporal-superior)	73.0 [66.4–79.7]
12 sector cpRNFL (superior-temporal)	81.6 [76.0–87.3]
12 sector cpRNFL (superior)	72.3 [65.5–79.1]
12 sector cpRNFL (superior-nasal)	74.1 [67.5–80.6]
12 sector cpRNFL (nasal-superior)	79.3 [73.2–85.3]
12 sector cpRNFL (nasal)	69.7 [62.6–76.7]
12 sector cpRNFL (nasal-inferior)	73.2 [66.3–80.1]
12 sector cpRNFL (inferior-nasal)	74.8 [68.2–81.4]
12 sector cpRNFL (inferior)	83.4 [78.0–88.9]
12 sector cpRNFL (inferior-temporal)	92.5 [88.9–96.1]
12 sector cpRNFL (temporal-inferior)	75.5 [69.0–81.9]
total m-RNFL	93.4 [90.0–96.8]
superior hemiretina mRNFL	80.2 [74.4–85.9]
inferior hemiretina mRNFL	91.4 [87.3–95.4]
64 grid m-RNFL	52.8 [44.9–60.7] – 94.3 [91.1–97.6]
total GCIPL	89.4 [85.2–93.6]
superior hemiretina GCIPL	79.4 [73.4–85.5]
inferior hemiretina GCLIPL	91.8 [88.2–95.4]
64 grid GCIPL	56.3 [48.4–64.2] – 91.2 [87.3–95.1]

AROC: Area Under the Receiver Operating Characteristic Curve, SD-OCT: spectral domain optical coherence tomography, CI: 95% confidence interval, cpRNFL: circumpapillary retinal nerve fiber layer (RNFL), mRNFL: macular RNFL, GCIPL: ganglion cell layer and inner plexiform layer combined.

Sensitivities at specificities of 80% and 90% were 100 and 98.8%, respectively for the Random Forests method; corresponding partial AROCs were 18.5 [CI: 16.8–19.6] and 14.4 [11.1–17.3] %. For the best single OCT parameter, sensitivities at specificities of 80% and 90% were 98.8 and 80.1%, respectively; corresponding partial AROCs were 8.5 [7.1–9.6] and 5.0 [CI: 2.5–7.4] %. Partial AROCs were significantly larger for the Random Forests method compared with the single best OCT parameter (P values = 0.018 and 0.005, respectively, after correction for multiple testing using Holm’s method [43], [44]).

For the Random Forests method, 83 of the 151 OCT measurements were significant predictors. Significant predictors included 360°-cpRNFL, mean, superior and inferior hemiretina mRNFL, mean, superior and inferioror hemiretina GCIPL, grid mRNFL in the inferior and superior temporal areas, grid GCIPL in the inferior and superior temporal areas as well as a number of 90°- and 30°-cpRNFL sectors: S(4), N(4), I(4), ST (12), SN (12), NS (12), I (12) and IT (12).

## Discussion

In the current study, 151 different SD-OCT measurements were captured in normal subjects and glaucoma patients, and the Random Forests method was applied to build a classifier of glaucoma based on these multiple measurements. As a result, a significantly larger AROC was obtained with the Random Forests method compared with any single OCT parameter.

The performance of classifiers at discriminating between normal subjects and glaucoma patients varies according to the stage of glaucoma investigated. As one would expect, the AROC tends to be smaller when early stage glaucoma eyes are included. [10], [15], [32] Tan et al. compared the OCT results of normal eyes and early glaucoma eyes (average MD of glaucoma eyes was equal to –4.6 dB) and reported an AROC of 90% with mean GCC thickness (mRNFL and GCIPL combined) over the macular area. [7] In addition, Kim et al. reported AROCs of 83.6 to 86.9% with whole circumference, and superior and inferior cpRNFL thickness measurements, and AROCs between 82.6 and 89.5% with total, and superior and inferior GCC thickness, in glaucoma eyes with an average MD of –8.49 dB. [12] In the current study, the average MD of glaucoma eyes was –5.6 dB, and the AROCs obtained using only cpRNFL or GCIPL averaged total or hemifield of the area were comparable with previous studies.

Early glaucomatous structural damage may start locally and hence may not be well represented in summary measures such as average thickness over quadrant cpRNFL or hemiretinal macular inner retinal layers. On the other hand, making a diagnosis based on SD-OCT parameters obtained from smaller regions may induce errors due to larger variability in the obtained results (attributable to increased measurement noise associated with smaller sample sizes). The limitations of measuring multiple SD-OCT parameters (captured over smaller regions) are somewhat controlled for by the Random Forests classifier as it can *concurrently* analyze all measurements recognizing global patterns and thereby reducing the problem of inherent measurement variability associated with analyzing a *single* OCT parameter [48], [49]; this appears to result in better discrimination between normal and pathologic eyes. In the current study, the AROC and partial AROCs obtained with the Random Forests method were significantly larger than those obtained using any single SD-OCT parameter. The sensitivity obtained was 93% with a specificity of 96%; this suggests that there is great potential for SD-OCT measurements, combined with the Random Forests method, to screen for glaucoma in a population-based or community-based setting.

In a previous study, Baskaran et al. [20] analyzed multiple OCT parameters using a decision tree method and reported an AROC of 98.2%; however, the average MD of glaucoma eyes in their study was –9.0 dB, which was much worse than that observed in the current study (average MD of our glaucoma eyes was –5.6 dB). Indeed, if we apply the decision tree method to our data, the AROC was equal to just 88.4%, significantly smaller than that obtained using the Random Forests method (using DeLong’s method after correction for multiple testing using Holm’s method [43], [44]). This result agrees with our previous study, which compared the decision tree method and Random Forests method to discriminate between perimetric glaucoma eyes and pre-perimetric glaucoma eyes. [23] Other studies have also investigated different statistical methods to classify glaucoma using SD-OCT measurements. Mwanza et al. analyzed multiple disc and SD-OCT parameters using logistic regression and reported an AROC of 97.4% in glaucoma eyes with an average MD of −3.2 dB. [18] Furthermore, Burgansky-Eliash et al. used a Support Vector Machine Classifier to analyze multiple TD-OCT parameters and reported that the AROC to discriminate normal and glaucoma eyes was as high as 98.1%. [19] The current Random Forests method concurrently analyzing cpRNFL, mRNFL and GCIPL parameters provided diagnostic ability that is at least comparable to that observed in these previous studies.

**Figure 4:**
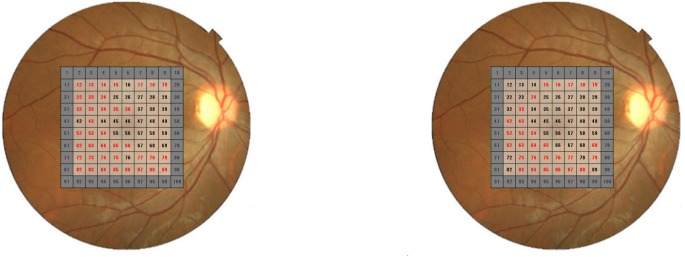
mRNFL (**Figure 4a**) and GCIPL grids (**Figure 4b**) that significantly contributed to the discrimination of glaucoma eyes from normal eyes (highlighted in red). mRNFL: macular retinal nerve fiber layer (RNFL), GCIPL: ganglion cell layer and inner plexiform layer combined.

Reports have suggested that the Random Forests method is more useful than other machine learning methods, such as support vector machines or boosting and bagging classifiers in predicting dementia, gene selection or fMRI decoding [27], [28], [50]. However, it is not known whether the Random Forests method outperforms other machine learning methods in discriminating glaucoma from normal eyes; this question awaits further research. Nevertheless a strength of the Random Forests method, in contrast to the support vector machine classifier, is its understandability and interpretability; the contribution of each parameter in the diagnosis process can be inferred, as shown in **Figure 4a, 4b**. Indeed, our results suggest that the superior and inferior-temporal peripapillary RNFLs and corresponding retinal area are likely to be involved in the early stage of glaucoma [16], [17] as these parameters significantly contributed to the discrimination between glaucoma and normal eyes.

Leave-one out cross validation was used to evaluate the performance of the Random Forests method in the current study. As described in the Methods section, the original dataset was divided into validation data (one patient) and training data (all other patients), and the Random Forests classifier is built using only training data of patients; this process was repeated so that every patient was used as a testing case once. Applying the classifier in the clinical setting is straightforward – a new patient can be classified according to the pre-determined optimum operating point (a 55.3% voting rate in the Random Forests classifier). Furthermore, the Random Forests classifier can be continuously trained by adding the data of new patients to it, which should further improve diagnostic accuracy. In addition, the Random Forests method carried out in this study was built using free statistical software and packages, specifically ‘R’, which is an open source statistical program (ver. 2.14.2; The R Foundation for Statistical Computing, Vienna, Austria).

One limitation of the current study is that eyes with high myopia (spherical refraction error <−6 diopters) were not included in the analysis. Many previous studies have indicated that the diagnostic ability of glaucoma imaging devices such as the Heidelberg Retina Tomograph (Heidelberg Engineering GmbH, Heidelberg, Germany) or OCT is worse in highly myopic eyes compared with near-emmetropic eyes [51]–[54]. Another limitation of the current study is that disc parameters were not included in the Random Forests classifier, which may have improved diagnostic performance; however, the 3D-OCT 1000 does not measure disc parameters such as rim area or vertical cup/disc ratio.

In summary, a combined analysis of cpRNFL-, mRNFL- and GCIPL-related thickness measurement using the Random Forests method was found to significantly improve the ability of SD-OCT to discriminate between normal and glaucoma eyes.

## Supporting Information

Data S1 **OCT data analyzed.**
(CSV)
